# Genome-Wide Analysis of WRKY Gene Family and Negative Regulation of *GhWRKY25* and *GhWRKY33* Reveal Their Role in Whitefly and Drought Stress Tolerance in Cotton

**DOI:** 10.3390/genes14010171

**Published:** 2023-01-07

**Authors:** Aiman Ehsan, Rubab Zahra Naqvi, Maryam Azhar, Muhammad Jawad Akbar Awan, Imran Amin, Shahid Mansoor, Muhammad Asif

**Affiliations:** Agricultural Biotechnology Division, National Institute for Biotechnology and Genetic Engineering (NIBGE), College of Pakistan Institute of Engineering and Applied Sciences (PIEAS), Jhang Road, Faisalabad 38000, Pakistan

**Keywords:** cotton, WRKY, drought, whitefly stress

## Abstract

The WRKY transcription factor family is marked by its significant responsiveness to both biotic and abiotic plant stresses. In the present study, the WRKY family of *Gossypium hirsutum* has been identified and classified into three groups based on the number of conserved WRKY domains and the type of zinc finger motif. This classification is further validated by conserved domain and phylogenetic analysis. Two members of the WRKY family, *WRKY25* and *WRKY33*, have been targeted through VIGS in *G. hirsutum*. VIGS-infiltrated plants were evaluated under drought stress and whitefly infestation. It was observed that *GhWRKY33*-downregulated plants showed a decrease in whitefly egg and nymph population, and *GhWRKY33* was found to be a strong negative regulator of whitefly and drought stress, while *GhWRKY25* was found to be a moderate negative regulator of whitefly and drought stress. As the targeted genes are transcription factors influencing the expression of other genes, the relative expression of other stress-responsive genes, namely *MPK6*, *WRKY40*, *HSP*, *ERF1*, and *JAZ1*, was also analyzed through qRT-PCR. It was found elevated in *GhWRKY33*-downregulated plants, while *GhWRKY25*-downregulated plants through VIGS showed the elevated expression of *ERF1* and *WRKY40*, a slightly increased expression of *HSP*, and a lower expression level of *MPK6*. Overall, this study provides an important insight into the WRKY TF family and the role of two WRKY TFs in *G. hirsutum* under drought stress and whitefly infestation. The findings will help to develop crops resilient to drought and whitefly stress.

## 1. Introduction

One of the most important crops in the agriculture sector, as well as the textile industry, is cotton (*G. hirsutum*). The cotton crop provides a wide range of products such as lint, oils, seed, and seed cake to various industries including the textile, food, and livestock industries. The cotton crop faces multiple challenges, and its cultivation requires high utilization of resources to protect the crop from damage by biotic and abiotic stresses and to produce better yield. There is a significant decline in cotton production worldwide due to multiple stress factors.

Among insect pests, the cotton whitefly (*Bemisia tabaci*) remains one of the most disastrous and notorious biotic threats to cotton. It causes damage to the cotton crop directly, indirectly, and through viral transmission. Nymphs and adults feed on plant sap by piercing, causing early wilting and stunting plant growth; this kind of damage is termed direct damage. Indirect damage includes honeydew excreted by whitefly that enhances the production of black sooty mildew and disrupts the photosynthesis of plants. Almost 40 diseases are caused by viral transmission through whitefly in cotton [1,2]. The most notorious and damaging viral disease of cotton is CLCuD (cotton leaf curl disease). Leaf curling disease of cotton is caused by viruses belonging to the genus *Begomovirus*. Collectively, these viruses cause a disease referred to as CLCuD, transmitted by whitefly. Sap-sucking insects have specialized piercing and sucking mouthparts. They inject saliva into plant tissues and suck back the phloem substance or sap of the plants. The sap of a plant is the liquid that maintains hydration and transports vital nutrients through the plant’s body. Therefore, sap-sucking insects can severely damage a plant by reducing its hydration and vital life-giving nutrients. Undigested sap is secreted and excreted, and it is called honeydew, due to having a high quantity of sugars. Its excess amount provides an environment for the growth of black sooty mildews, and hence, photosynthesis of leaves is disrupted. This is termed indirect damage [3].

Water deficiency or drought is one of the major abiotic stresses that negatively affect crop yield and fiber quality in cotton. Water is the main component of a cell and plays a major role in various stages of the life cycle of cell-like growth, development, nutrient transport, cellular reactions, transpiration, and photosynthesis. Any irregularity in water availability adversely affects the overall functioning of the plant and jeopardizes its survival. Short-term water deficiency causes stunted growth, while long-term drought conditions cause wilting and death [4]. The primary signal generated during drought stress is hyperosmotic stress. Hyperosmotic stress causes the release of water from cells and the shrinkage of cells. Moreover, it causes the accumulation of abscisic acid in cells, which activates drought-stress-related genes. The secondary effects of drought stress are complex and include damage to cellular components, oxidative stress, and metabolic malfunctioning. Most of the plant responses to drought stress arise due to secondary signals. Different parameters affected by drought stress include overall plant height, root and shoot growth, photosynthesis, relative water content, yield, fiber quality, and vegetative and reproductive growth [5].

Under stress conditions, plants respond by altering the expression of their signaling cascades through transcription factors. The WRKY TF gene family is one of the ten largest families of transcription factors in plants that regulate the expression of various pathways of PTI and ETI, both positively and negatively; hormonal signaling pathways; growth; and stress response [6]. The WRKY transcription factor family derived its name from a generally conserved domain of WRKYGQK amino acid sequence at the *N*-terminal. However, some other variants, such as WSKY, WRRY, WVKY, WKRY, and WKKY, are also found rarely in WRKY proteins. Another conserved feature of the WRKY transcription family is a novel zinc finger motif (CX4–5CX22–23HXH or CX7CX23HXC) at the *C*-terminal [7,8]. WRKY transcription factors form homo- or heterocomplexes and recognize a specific *cis*-regulatory element (5′-TTGAC(C/T)-3′) of the promoter region of a gene called W-box through an almost 60-amino-acid-long chain at the *N*-terminal [9]. Major contact between DNA and WRKY protein occurs through conserved WRKYGQK interaction with thymine methyl groups of W-box [10]. The β-sheet of a WRKY TF horizontally enters into the major groove of the B form of DNA and establishes apolar contacts. Mainly, tryptophan (W), tyrosine (Y), and two lysine (K) residues of the WRKYGQK domain are crucial for developing a stable interaction [11,12,13,14].

WRKYs are classified into three groups, Groups I, II, and III, based on the number of WRKYGQK conserved domains and the type of zinc finger motif [15]. Group I of WRKYs has two conserved WRKY domains, and Groups II and III have one domain. Group II is further subdivided into IIa, IIb, IIc, IId, and IIe. WRKYs of Groups II and III are differentiated by the structure of zinc finger nuclease. Groups I and II of WRKYs have a C_2_H_2_ (CX_4–5_CX_22–23_HXH) zinc finger domain, while Group III WRKYs have a C_2_HC (CX_7_CX_23_HXC) zinc finger domain [16]. Stress conditions may induce or suppress WRKY expression, and also, WRKYs respond positively or negatively to stress conditions. Some WRKYs work in favor of stress, while some work against it through a complex signaling network [17,18]. Expression and accumulation of WRKY mRNA is highly cell-specific and held under strict physiological conditions, and sometimes, it does not even translate into proteins. Some WRKYs play a dual role, acting as either positive or negative regulators of stress in defense signaling [19]. Some WRKYs form homo- and heterodimers, which influence DNA binding capability. In promoter regions, the majority of WRKYs have multiple *cis*-regulatory W-box elements, and they can regulate their expression by themselves and also be cross-regulated by other WRKYs; hence, WRKY TFs work through mutual manipulation channels [20,21].

Transcriptome analysis reveals that the expression of WRKYs is highly variable and tissue-specific. Most of the Group I and II WRKYs were found to be involved during growth and fiber elongation, senescence, and aging, while Group-III WRKYs were considered to be involved during stress conditions. Transcriptome levels and expression of WRKYs are tightly regulated, and they rapidly change under different kinds of biotic and abiotic stresses [22,23]. As a whole, like in other crops, WRKYs play a widespread role in *G. hirsutum*, especially under stress conditions, but there is a lot more to explore and investigate about the complex crosstalk of this gene family [24,25].

To analyze the function of a gene under a specific stress condition, a reverse genetics approach is utilized. Virus-Induced Gene Silencing (VIGS) is a tool of reverse genetics developed by utilizing a natural defense mechanism of plants against viruses called post-transcriptional gene silencing (PTGS) [26]. VIGS is developed as a tool to induce transient PTGS by using viruses as a vector to carry a fragment of the target gene [27]. For VIGS experimentation, the Ti region of a viral vector is genetically modified to contain a fragment of the target gene and inoculated into plants via *Agrobacterium tumefaciens*. Upon inoculation, viral machinery replicates the target gene fragment, which spreads systemically in the plant, and dsRNA intermediates are produced [28]. These intermediates trigger the RNA-mediated degradation of a target gene in the host plant [29].

In this study, we performed a genome-wide analysis and classification of the WRKY family in *G. hirsutum.* The classification of the WRKY gene family was further confirmed by conserved domain analysis and phylogenetic analysis. The upstream 1000 bp promotor region of selected genes *GhWRKY25* and *GhWRKY33* was also analyzed for the presence of promotor motifs. It has been previously established that *GhWRKY25* and *GhWRKY33* act as negative regulators of drought and enhance drought sensitivity. Their overexpression under drought stress results in inhibited root growth, lower chlorophyll and proline, a decreased ratio of stomata length to width, and reduced sensitivity to abscisic acid [30,31]. This indicates that *WRKY25* and *WRKY33* silencing can generate tolerance against drought stress. Furthermore, both these transcription factors are also reported to be negative regulators of the SA pathway during a pathogen attack [30,32,33]. Therefore, *GhWRKY25* and *GhWRKY33* were chosen further for functional validation under drought and whitefly stress through VIGS assay in cotton plants. The functional analysis of *GhWRKY25* and *GhWRKY33* was performed by keeping VIGS-infiltrated plants under drought stress and whitefly infestation while keeping non-infiltrated and empty-plasmid-infiltrated plants as control. The relative expression of whitefly-responsive genes such as *MPK6*, *WRKY40*, *HSP*, *ERF1*, and *JAZ1* was also analyzed. The study provides a comprehensive analysis of the WRKY gene family and its role in drought and whitefly resistance in cotton.

## 2. Materials and Methods

### 2.1. Classification and Characterization of WRKYs

All available WRKY sequences were retrieved from CottonGen assembly *G. hirsutum* AD1 ‘NDM8’ HEAU v1.0 Genome (26 chrom. and 227 scaffolds) via BLAST of one known WRKY sequence and obtained hits in the assembly. The retrieved sequences were aligned through ClustalW multiple sequence alignment. The WRKY proteins were evaluated by calculating their isoelectric point, molecular weight, number of amino acids, instability index, aliphatic index, and hydropathicity through ExPASy ProtPram. The conserved domain analysis was performed with the help of MEME suite by using the standard settings, and the number of motifs was selected as 10. The phylogenetic analysis was performed using MEGA6 software [34]. A maximum likelihood tree was constructed by keeping the bootstrap value at 1000. For promotor analysis, 1000 bp upstream region was retrieved from cottonFGD, and promotor motifs were found through PlantCARE.

### 2.2. Construction of TRV-Based Vectors

RNA of young *G. hirsutum* leaves was extracted and converted to cDNA by using cDNA synthesis kit (Thermofisher Scientific, Waltham, MA, USA, Cat No. K1621). Selected regions were amplified through PCR, and primers were designed on the regions given by the SGN-VIGS tool through Prime3. Plasmid and amplicon were restricted through *EcoR*I and *Kpn*I to produce sticky ends. T4 ligase (Thermo Scientific, Cat No. EL0011) with its specific ligation buffer (10X) was used for ligation. The ligation product was used for the transformation of *Escherichia coli* chemical competent cells. Clones were named as TRV2-WRKY25 and TRV2-WRKY33 by considering the cloned fragment of the targeted gene. Clones were subjected to PCR-based screening, and primers from Li et al., [35] were used. From positive clones, the plasmid was isolated and restricted with *EcoR*I and *Kpn*I to release ligated fragments. GV3101 *A. tumefaciens* competent cells were transformed with positive TRV constructs, colonies were subject to PCR-based screening, and primers from Table 1 were used.

### 2.3. Plant Growth Conditions

Ginning of cotton variety coker 312 was performed, followed by de-linting by washing with H_2_SO_4_. Seeds were sown in peat mix in plastic glasses. The plants were grown until the cotyledon stage for infiltration [36]. After infiltration, plants were again kept in greenhouse, and the environmental conditions were kept at 30 ± 2 °C day/24 ± 2 °C night for a 16 h day length.

### 2.4. Agrobacterium-Based Infiltration

Agrobacterium cultures of TRV1, TRV2, TRV2-GrCla, TRV2-WRKY25, and TRV2-WRKY33 were grown from a single colony in LB medium supplemented with 50 μg/mL of Kanamycin and 25 μg/mL of Rifampicin overnight at 28 °C. The above culture was transferred to a flask with 50 mL of LB medium supplemented with 50 μg/mL of Kanamycin and 25 μg/mL of Rifampicin, plus 10 mM MES and 20 μM acetosyringone. After overnight incubation at 28 °C, agrobacterial cells were palleted at 4000 rpm for 5 min and re-suspended in the infiltration buffer containing 10 mM MgCl_2_, 10 mM MES, and 200 μM acetosyringone. The OD of the culture was maintained at 1.5, and the culture was left on the bench at room temperature for 3 h. Agrobacterium culture suspension of TRV1 was mixed with agrobacterium culture suspension of TRV2, TRV2-GrCla, TRV2-WRKY25, and TRV2-WRKY33 in a 1:1 ratio; the mixture was infiltrated from the underside of cotyledons through the wounding sites using a 1 mL needleless syringe. After infiltration, the plants were kept in greenhouse under controlled conditions (30 ± 2 °C day/24 ± 2 °C night at a 16 h day length) for 10 days until photobleaching appeared in true leaves of TRV2-GrCla-infiltrated plants.

### 2.5. RNA Isolation and cDNA Synthesis

After photobleaching started appearing in true leaves of TRV2-GrCla-infiltrated plants, total RNA was isolated from true leaves of non-infiltrated control, TRV:00-, TRV2-WRKY25-, and TRV2-WRKY33-infiltrated plants. RNA extraction protocol recommended by the supplier (Invitrogen, Carlsbad, CA, USA) of RNA extraction kit TRIzol Plant RNA isolation reagent (Invitrogen) was used for the isolation of total RNA from cotton leaves. RNA samples were quantified followed by treatment with DNase to remove any traces of plant genomic DNA. The cDNA was prepared from RNA using RevertAid First-strand cDNA Synthesis Kit (Thermo Fisher Scientific, Cat No. K1621).

### 2.6. Real-Time Quantitative PCR Based Expression Analysis

Real-time quantitative PCR (qPCR) analyses were performed to verify the downregulation of targeted genes and evaluate the difference in expression of genes from pathways of targeted genes in non-infiltrated, TRV:00-, TRV:TRV2-WRKY25-, and TRV:TRV2-WRKY33-infiltrated cotton plants. Primers from Table 1 were used for qPCR analysis. Relative expression of some other whitefly-responsive genes (*MPK6*, *WRKY40*, *HSP*, *ERF1*, *JAZ1*) [35] from non-infiltrated and infiltrated cotton plants was also analyzed via qRT-PCR after the confirmation of downregulation of targeted gene. The qPCR was performed by using the SYBER green real-time PCR master mix (Thermo-Fisher Scientific). The cDNA was used as the template. The reactions were performed in triplicates. The profile was set as 95 °C for 5 min, followed by 40 cycles of 95 °C for 30 s; 55 °C for 30 s, and 72 °C for 30 s. A melt curve analysis was conducted following the completion of the amplification cycles. Through the ΔΔCt method, relative fold difference for each sample in each experiment was calculated. The UBQ7 gene was used as an internal control to normalize the results.

### 2.7. Drought Assay

Infiltrated *G. hirsutum* plants (TRV:TRV2-WRKY25-, TRV:TRV2-WRKY33-, and TRV:00-infiltrated) and non-infiltrated plants were kept while withholding water. The water was withheld for 30 days from the very next day after observing photobleaching of the TRV:Grcla VIGS-infiltrated cotton plants. Phenotypic changes in leaf were observed.

### 2.8. Whitefly Bioassay

Infiltrated *G. hirsutum* plants (TRV:TRV2-WRKY25-, TRV:TRV2-WRKY33-, and TRV:00-infiltrated) and non-infiltrated plants as another set were kept in whitefly infestation under control conditions. Production of eggs and nymphs was counted from small sections of leaves under a magnifying glass. Data on egg and nymph numbers were recorded at 7th and 15th days of whitefly infestation. Analysis of variance (ANOVA), simple sample *t*-test, and LSD test were performed on the data.

## 3. Results

### 3.1. Classification of GhWRKYs through Multiple Sequence Alignment

**Figure 1:**
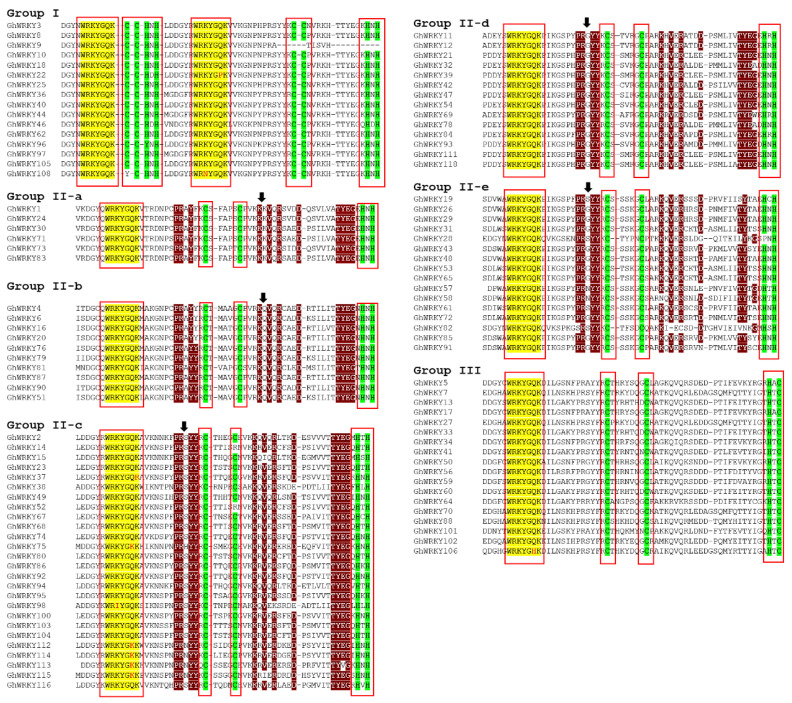
Multiple sequence alignment of amino acids of *G. hirsutum* WRKY sequences. The typical amino acids in the WRKY domain are represented in yellow color, substitutions in the WRKY domain are represented by red letters, and amino acids in the zinc finger motif are represented in green color. Maroon color represents the amino acid sequences on which classification of Group II WRKYs was performed.

All possible *G. hirsutum* WRKY sequences were retrieved from CottonGen assembly *G. hirsutum* AD1 ‘NDM8’ HEAU v1.0 Genome (26 chrom. and 227 scaffolds). We identified 107 WRKYs in the cotton genome that have a conserved WRKYGQK domain at the C-terminal followed by a zinc finger motif at the *N*-terminal. Some WRKYs have single or double amino acid substitutions in the WRKYGQK domain; for instance, one WRKY domain of *GhWRKY22* has WRKYGPK, and *GhWRKY108* has WRNYGQK. *GhWRKY37* and *GhWRKY98* also have single amino acid substitutions: *GhWRKY37* has WRKYGQR, and *GhWRKY98* has WRIYGQK. Some other WRKYs such as *GhWRKY112*, *GhWRKY113*, *GhWRKY114*, and *GhWRKY115* have double amino acid substitutions. These 107 GhWRKYs were classified as Groups I, II, and III based on the number of WRKY domains and type of zinc finger motif (Figure 1). The Groups I WRKYs have two WRKY domains either at the C- or *N*-terminal of the protein and a C-X_n_-C-X_n_-HXH-type zinc finger motif at the *N*-terminal of each WRKY domain. *GhWRKY9*, a member of Group I, has only one zinc finger motif at the *N*-terminal of one of its two WRKY domains. Group II WRKYs have one WRKY domain at the C-terminal and one C-X_n_-C-X_n_-HXH-type zinc finger motif at the *N*-terminal of the WRKY domain. Group III WRKYs have one WRKY domain at the C-terminal and one C-X_n_-C-X_n_-CXH-type zinc finger motif at the *N*-terminal of the WRKY domain. The Group II GhWRKYs were further subdivided into II-a, II-b, II-c, II-d, and II-e based on the difference in conserved amino acid sequences by keeping AtWRKYs from each group as reference. Group I was found to have 16 GhWRKYs, Group II-a has 6, II-b has 10, II-c has 26, II-d has 14, II-e has 16, and Group III has 18 WRKYs. The classification of WRKYs identified here in cotton is shown in Figure 1, and the specific parameters such as PI, M/W, no. of amino acids, instability index, aliphatic index, and hydropathicity are given in Supplementary Table S1. In the Group I WRKYs, PI ranges from 7–9, M/W from 35,000–75,000, number of amino acids from 250–750, instability index from 40–60, aliphatic index from 40–70, and hydropathicity from −0.7 to −0.9. In the Group II WRKYs, PI ranges from 4–9.5, M/W from 15,000–150,000, number of amino acids from 150–950, instability index from 25–75, aliphatic index from 40–120, and hydropathicity from −1.1 to 0.1. In the Group III WRKYs, PI ranges from 5–9, M/W from 20,000–40,000, number of amino acids from 200–500, instability index from 40–65, aliphatic index from 45–70, and hydropathicity from −0.9 to −0.4. Overall, the results show that 107 GhWRKYs were classified as Groups I, II, and III based on the number of WRKY domains and type of zinc finger motif.

### 3.2. Conserved Domain Analyses

**Figure 2:**
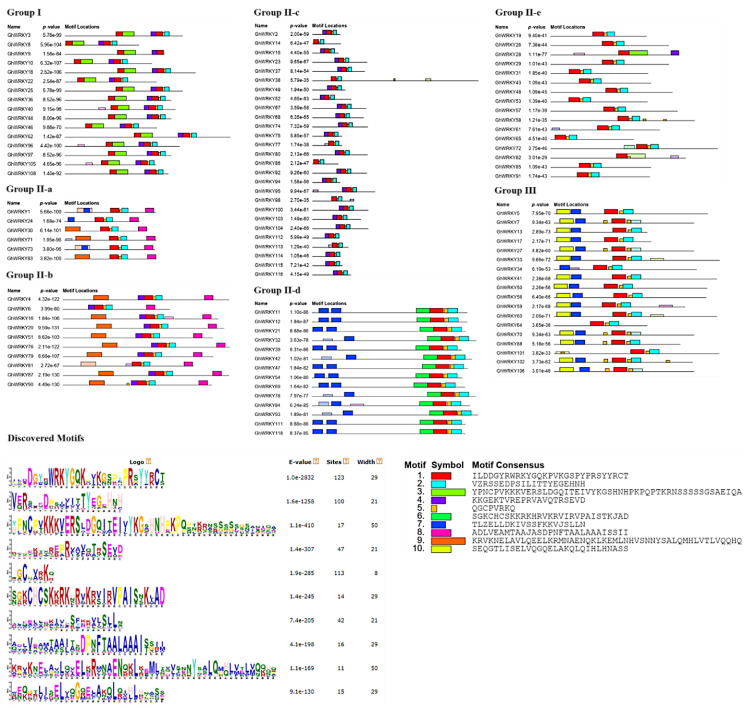
Group-wise conserved domain analysis of GhWRKYs. Conserved domain analysis is performed through MEME suite. The discovered motifs are assigned a specific color and length by which they are represented in the sequence. The details of the individual motifs were found.

For a better understanding of the conservation and diversity of WRKYs, conserved domain analysis was performed through the MEME suite. The system was commanded to show ten conserved motifs. The results show that the WRKYs that were placed in one group showed a closely similar pattern of conserved domains. Domain 1 represents the WRKY domain, and Domain 2 represents the zinc finger motif; these two domains are present in all sequences. Domain 3 is only present in Group I WRKYs and one WRKY (*GhWRKY28*) of Group II-e as an exception. Domain 4 is present in Groups I, II-b, and II-c. Domain 5 is quite common and present in many of the sequences of all groups. Domain 6 is only present in Group II-d. In Group II-d, Domain 7 is present twice in almost every sequence, while in Group III, Domain 7 is present only once. It was also present in some sequences of Group II-a. Domains 8 and 9 are present in only Groups II-a and II-b, while Domain 10 is only present in Group III. This conserved domain analysis represents the similarity in the amino acid sequences within the groups and the difference in sequences among the groups. The distribution of ten identified conserved domains in three classes of GhWRKYs is shown in Figure 2.

### 3.3. Phylogenetic Relationship

**Figure 3:**
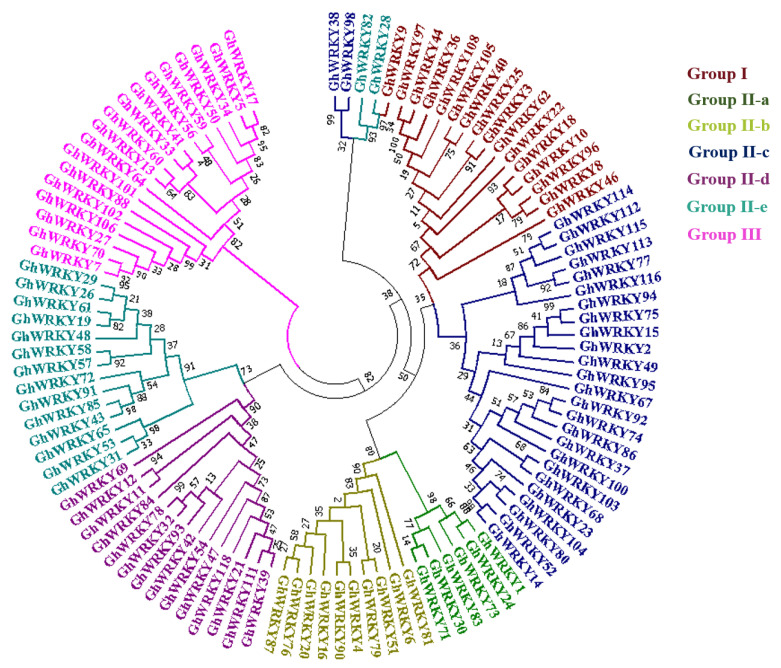
Phylogenetic analysis of WRKY proteins. For phylogenetic analysis of *G. hirsutum*, the tree is drawn to scale, with branch lengths measured in the number of substitutions per site. The maximum likelihood tree includes WRKY protein sequences of *G. hirsutum.* Each group of GhWRKYs is represented by a unique color.

For phylogenetic analysis, a maximum likelihood tree was constructed for WRKY protein sequences of *G. hirsutum* through MEGA6 (Figure 3). In the phylogenetic tree, each group is shown as a separate clade and represented by a different color. Group I is represented by maroon color, Group II-a is represented by green color, Group II-b is represented by yellow color, Group II-c is represented by blue color, Group II-d is represented by purple color, Group II-e is represented by cyan color, and Group III is represented by pink color. The tree shows that Group I is closely related to Group II-c. Group II-a and II-b also show close relations to Group I and Group II-c. Group II-d and Group II-e are very close to each other while being significantly different from other groups. Group III is significantly different from all other groups.

### 3.4. Promotor Analysis of WRKY25 and WRKY33

For the genes selected to be analyzed by VIGS, promotor analysis was performed at a 1000 bp upstream region through PlantCARE. Most of the motifs were common in the promotor region of both genes, while some were unique to each. W-box motif, which is recognized by WRKY TF to regulate gene expression, was found in the promotor region of *WRKY33* but not in the promotor region of *WRKY25.* Some other motifs such as abscisic-acid-responsive motif (ABRE), light-responsive motif (ATCT, GT1-motif), and cis-acting regulatory elements (ARE, CAT-box, TCA-element) were absent in the promotor region of *WRKY25.* Some motifs such as gibberellin-responsive P-Box and Myb were absent in the promotor region of *WRKY33.* A core promotor element of transcription TATA box and a common cis-acting element CAAT-box were found abundantly in the promotor region of both genes. Each promotor motif along with its sequence, function, and no. of repeats present in the promoter region is presented in the Table 2.

### 3.5. Development of TRV-Based Constructs for GhWRKY25 and GhWRKY33

**Figure 4:**
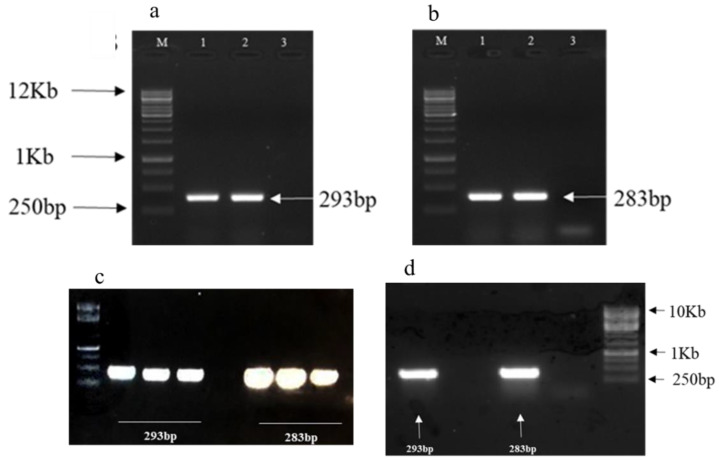
PCR amplification results of (**a**) 293 bp band of *WRKY25* amplified from *G. hirsutum* cDNA and (**b**) 283 bp band of *WRKY33* amplified from *G. hirsutum* cDNA, (**c**) 293 bp band of *WRKY25* and 283 bp band of *WRKY33* amplified from grown cultures of *E. coli* transformed cells, indicating the presence of recombinant TRV2 vector, (**d**) 293 bp band of *WRKY25* and 283 bp band of *WRKY33* amplified from grown cultures of transformed cells of *GV3101*, indicating the presence of recombinant TRV2 vector.

For the VIGS assay, TRV-based VIGS constructs were developed by using the amplified products of *GhWRKY25* and *GhWRKY33* from cDNA of *G. hirsutum*. PCR for screening of positive plasmids yielded a 293 bp band of *WRKY25* and a 283 bp band of *WRKY33*, confirming the positive clones with the desired plasmid in *E. coli* and *Agrobacterium GV3101* ( (Figure 4).

### 3.6. VIGS-Based Functional Analysis of GhWRKY25 and GhWRKY33 under Drought Stress and Whitefly Infestation

#### 3.6.1. Downregulation of Targeted Genes

**Figure 5:**
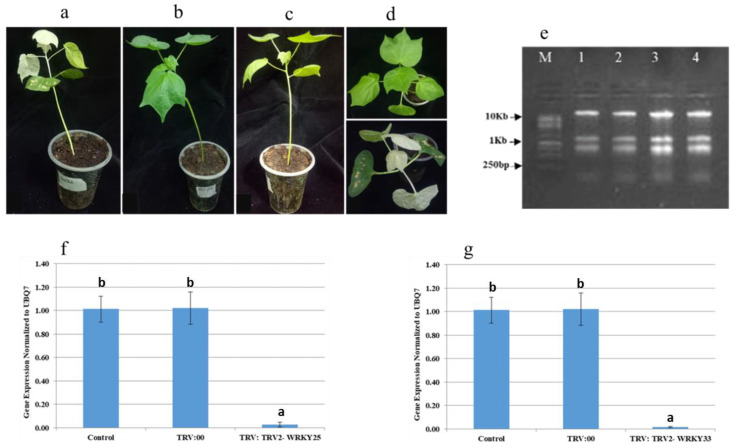
VIGS-based functional analysis of GhWRKY25 and GhWRKY33 genes in cotton. (**a**–**d**) Photobleaching appeared in TRV:TRV2-GrCla-infiltrated *G. hirsutum* plants, while no phenotypic symptom appeared in TRV:TRV2-WRKY25- and TRV:TRV2-WRKY33-infiltrated plants. (**e**) Total RNA of 1. non-infiltrated and 2–4. VIGS-infiltrated plants; M shows 1 Kb DNA Marker. (**f**) Quantitative real-time expression analysis of *WRKY25* normalized against expression level of *UBQ7*. RNA samples were extracted from leaves of control non-infiltrated, TRV:00-infiltrated, and TRV:TRV2-WRKY25-infiltrated cotton plants. Error bars indicate deviation between three experimental repeats. y-axis shows the relative gene expression in plants mentioned on x-axis. (**g**) Quantitative real-time expression analysis of *WRKY33* normalized against expression level of *UBQ7*. RNA samples were extracted from leaves of control non-infiltrated, TRV:00-infiltrated, and TRV:TRV2-WRKY33-infiltrated cotton plants. Error bars indicate deviation between three experimental repeats. y-axis shows the relative gene expression in plants mentioned on x-axis. Specific letters are assigned to bars according to a standard error within replicates.

Ten days after infiltration, photobleaching appeared in true leaves of TRV:TRV2-GrCla-inoculated plants, and photobleaching was rendered as a marker for the onset of silencing (Figure 5). The TRV1:TRV2-WRKY25- and TRV1:TRV2-WRKY33-infiltrated plants did not show any phenotypic alternation in their true leaves. Therefore, the qRT-PCR-based expression analysis of *WRKY25* and *WRKY33* from RNA of VIGS-infiltrated plants was performed, and it revealed the downregulation of targeted genes.

#### 3.6.2. Drought Bioassay

**Figure 6:**
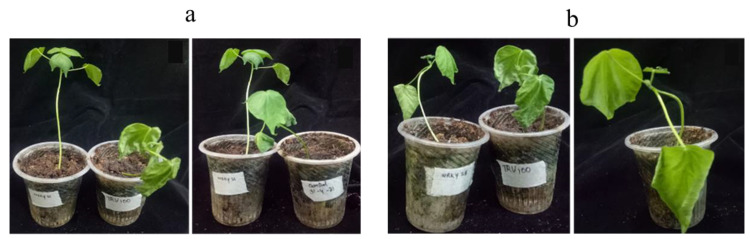
Drought bioassay on cotton VIGS-infiltrated plants. (**a**) Non-infiltrated, TRV:TRV2-WRKY33-, and TRV:00-infiltrated cotton plants at 30th day of drought stress. (**b**) TRV:TRV2-WRKY25- and TRV:00-infiltrated cotton plants at 30th day of drought stress.

VIGS-infiltrated and non-infiltrated cotton plants were kept under drought stress after confirmation of targeted gene downregulation by withholding water. TRV:TRV2-WRKY25-infiltrated plants showed wilting symptoms after 2 weeks, while TRV:TRV2-WRKY33-infiltrated plants showed wilting symptoms after one month. TRV:00-infiltrated plants showed wilting symptoms after one week, while non-infiltrated plants showed wilting symptoms after 7–10 days (Figure 6). Upon re-watering, gene-downregulated plants recovered, while TRV:00 and non-infiltrated plants failed to recover and showed permanent wilting and also yellowing. *GhWRKY33* was found to be a strong negative regulator of drought stress, while *GhWRKY25* was observed to be a moderate negative regulator of drought stress.

#### 3.6.3. Whitefly Bioassay

**Figure 7:**
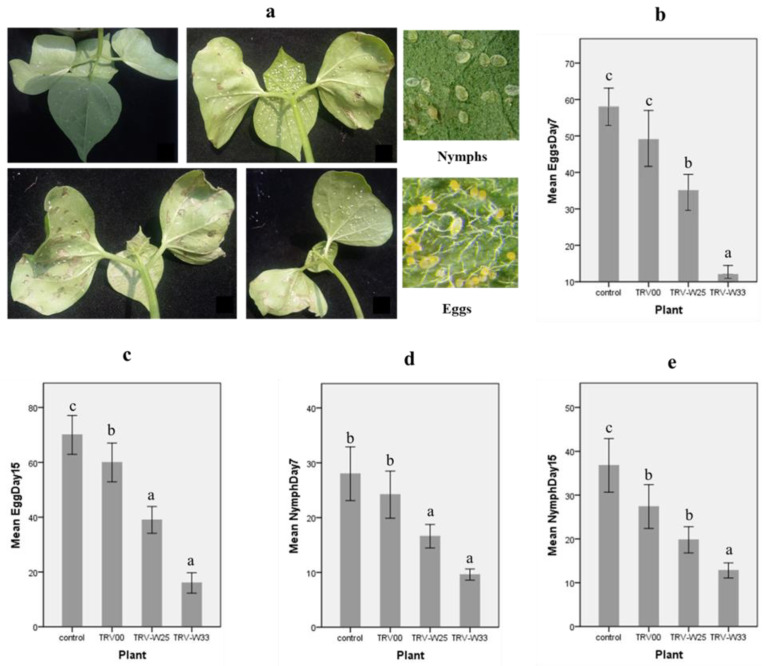
Whitefly bioassay on cotton VIGS-infiltrated plants. (**a**) Non-infiltrated, TRV:TRV2-WRKY33-, TRV:TRV2-WRKY25-, and TRV:00-infiltrated cotton plants under whitefly infestation. (**b**) Colored bars represent egg production at 7th day of whitefly infestation. Error bars show standard error within the replicates. Specific letters are assigned to bars according to a standard error within replicates, with a 95% level of the confidence interval and a *t* value of 9.235. (**c**) Colored bars represent egg production at 15 days of whitefly infestation. Error bars show standard error within the replicates. Specific letters are assigned to bars according to a standard error within replicates, with a 95% level of the confidence interval and a t value of 9.352 (**d**) Colored bars represent nymph production at 7th day of whitefly infestation. Error bars show standard error within the replicates. Specific letters are assigned to bars according to a standard error within replicates, with a 95% level of the confidence interval and a *t* value of 10.829 (**e**) Colored bars represent nymph production at 15th day of whitefly infestation. Error bars show standard error within the replicates. Specific letters are assigned to bars according to a standard error within replicates, with a 95% level of the confidence interval and a *t* value of 10.647.

Data for number of nymphs and eggs from small sections of 1 mm^2^ from different leaves were collected by using magnifying glass at 7th day and 15th day. Average number of nymphs and eggs, standard error, and significant differences within the replicates of data at different stages were represented in the form of bar graph diagrams. Bar graphs show that TRV:TRV2-WRKY33-infiltrated plants showed visibly reduced nymph and egg production. On non-infiltrated and TRV:00-infiltrated cotton plants, nymph and egg production was abundant. The non-infiltrated and TRV:00-infiltrated plants were used as control, and the production of eggs and nymphs on leaves of infiltrated plants was compared with control plants. On the 7th day, the whitefly egg production was 80% less than that of non-infiltrated plants, and on day 15th, it was 78% less in WRKY33-downregulated plants. The nymph production on the 7th and the 15th days was 67% less than that of the non-infiltrated control plants. The TRV:TRV2-WRKY25 showed a small reduction in the production of nymph and eggs on the 7th and 15th day, and the whitefly egg and nymph production was on average 35–40% less than that of non-infiltrated control plants at all stages. In *WRKY33*-downregulated plants, the mean number of whitefly eggs was 12 at day 7 and 16 at day 15, and the mean number of whitefly nymphs was 9 at day 7 and 12 at day 15. In *WRKY25*-downregulated plants, the mean number of whitefly eggs was 35 at day 7 and 39 at day 15, and the mean number of whitefly nymphs was 16 at day 7 and 19 at day 15. As compared to non-infiltrated control, the mean number of whitefly eggs was 58 at day 7 and 70 at day 15, and the mean number of whitefly nymphs was 28 at day 7 and 36 at day 15 (Figure 7). The pattern of growth remained almost same after the 15th day. In a nutshell, *GhWRKY33*-downregulated plants showed a decrease in whitefly egg and nymph population and was found to be a strong negative regulator under whitefly infestation, whereas *GhWRKY25* was found to be a moderate negative regulator of whitefly infestation stress.

#### 3.6.4. Relative Expression of Other Whitefly-Responsive Genes

**Figure 8:**
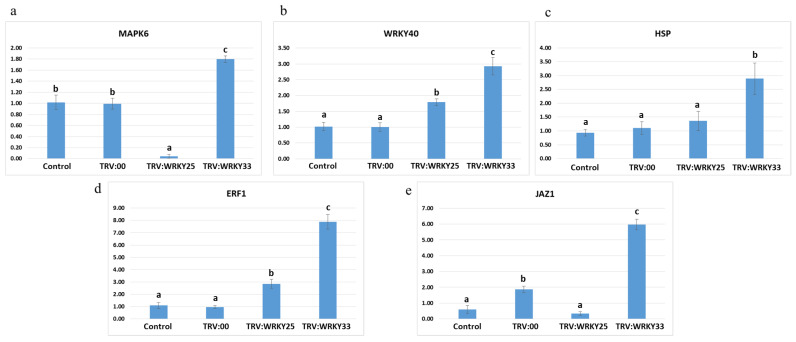
Relative expression analysis of *MPK6*, *WRKY40*, *HSP*, *ERF1*, *JAZ1* in VIGS-infiltrated cotton plants. Quantitative real-time expression of *MPK6, WRKY40, HSP, ERF1, JAZ1* is shown from (**a**–**e**) in leaves of non-infiltrated, TRV:00-infiltrated, TRV:TRV2-WRKY25-, and TRV:TRV2-WRKY33-infiltrated cotton plants. Error bars indicate deviation between three experimental repeats, and specific letters are assigned to bars according to a standard error within replicates.

The relative expression analysis of whitefly-responsive genes *MPK6*, *WRKY40*, *HSP*, *ERF1*, and *JAZ1* in infiltrated and non-infiltrated plants revealed a remarkable difference. *MAPK6* and *JAZ1* were found upregulated in *WRKY33*-downregulated plants while being downregulated in *WRKY25*-downregulated plants. *WRKY40* was found slightly upregulated in *WRKY25*-downregulated plants while being remarkably upregulated in *WRKY33*-downregulated plants, and the pattern was almost same for HSP and ERF1 (Figure 8).

## 4. Discussion

WRKY family is involved in the regulation of many biotic and abiotic stresses [17]. In this study, we have executed a genome-wide analysis of the WRKY gene family in cotton, and our classification corroborates with previous studies [37]. In some previous studies, WRKY TFs have been classified in various crops such as rice, wheat, barley, and cotton (*G. raimondii*) by considering the classical classification of *Arabidopsis* as reference. In this study, the classification was created for *G. hirsutum* by considering the previously created classifications of various crops. The classification was further confirmed by conserved domain analysis and phylogenetic analysis. Each group showed an almost similar pattern of conserved domains and made a separate cluster in the phylogenetic tree. Just like the previous studies, WRKY TFs of *G. hirsutum* were found to be categorized into three groups. In Group I, the sequences having two WRKY domains at C-terminal and one C-X_n_-C-X_n_-CXC-type zinc finger motif at the *N*-terminal were placed. In Group III, the WRKY sequences having one WRKY domain at the C-terminal and one C-X_n_-C-X_n_-CXH-type zinc finger motif at the *N*-terminal of the WRKY domain were placed. In Group II, sequences with one WRKY domain at C-terminal and one C-X_n_-C-X_n_-CXC-type zinc finger motif at the *N*-terminal were placed. Group II WRKYs were further subdivided into II-a, II-b, II-c, II-d, and II-e based on the difference in conserved amino acid sequences. Conserved domain analysis further confirmed this classification and showed a similar pattern of conserved motifs in each group. These findings are similar to those of classification in other crops. The calculation of isoelectric point, molecular weight, number of amino acids, instability index, aliphatic index, and hydropathicity of WRKY proteins in *G. hirsutum* revealed that Group II WRKY proteins have larger diversity than the other two groups because of larger size. For this study, *GhWRKY25* and *GhWRKY33* were selected to target through VIGS to explore their role under drought and whitefly stress. Promotor sequence analysis of a 1000 bp upstream region revealed that there is a striking difference in diversity and quantity of promotor motifs in the promotor region of both genes. The W-box motif, which is recognized by WRKY TF to express or repress the gene expression, was found in the promotor region of *GhWRKY33* gene but absent in the promotor region of *GhWRKY25.* That means *WRKY33* can be expressed or suppressed via other TFs of WRKY family. Another promotor motif is TCA-element, which is a cis-acting element involved in salicylic acid responsiveness. It was found in the promotor region of WRKY33 gene, indicating its responsiveness to salicylic acid bio-signaling, but it was found absent in the promotor region of *WRKY25*. It has been reported that overexpression of the two WRKY TFs *WRKY25* and *WRKY33* shows enhanced susceptibility to drought [30,31]. In the present study, we have performed functional analysis of these genes through VIGS in *G. hirsutum.* After silencing the genes, the plants were kept under drought and whitefly stress. Under drought stress, *GhWRKY33*-downregulated plants remain intact even after 30 days, which showed *GhWRKY33* is a strong negative regulator of drought stress. *GhWRKY25*-downregulated plants resisted drought stress for a while, but soon, they started showing wilting, which shows *GhWRKY25* might have some small role in regulating drought stress. Another interesting thing was that *GhWRKY33*- and *GhWRKY25*-downregulated plants flourished again after rewatering (40 and 30 days after drought stress, respectively) but non-infiltrated and TRV0-infiltrated plants showed permanent wilting. Under whitefly stress, *GhWRKY25*-downregulated plants showed a slight decrease in the production of eggs and nymphs of whitefly. Results provide some shreds of evidence for *GhWRKY25* to be a mild negative regulator of whitefly stress. Another WRKY TF *GhWRKY33*, a negative regulator of drought stress when subjected to whitefly stress in the current study, was found to be a negative regulator of whitefly stress as well. Under whitefly stress, *GhWRKY33*-downregulated VIGS-infiltrated plants showed reduced production of eggs and nymphs of whitefly. Results indicate silencing of *WRKY33* made cotton plants tolerant to whitefly stress. Therefore, *GhWRKY33* is considered to be involved in the enhancement of susceptibility to whitefly.

Another important insight we obtained from this study was that there is a significant alternation of expression of other whitefly-responsive genes in non-infiltrated and infiltrated plants. The qPCR primers to check the expression of whitefly-responsive genes *MPK6*, *WRKY40*, *HSP*, *ERF1*, and *JAZ1* were taken from a previous study [35]. MPK6, a stress-responsive gene [38] that also interacts with whitefly effectors [39], was found downregulated in *GhWRKY25*-downregulated plants, while its expression was elevated in *GhWRKY33*-downregulated plants. This shows *GhWRKY25* is required for the expression of *MPK6*, while *GhWRKY33* acts as a suppressor of *MPK6*. *WRKY40*, which was found to be an important component of regulating resistance to whitefly stress [35], was found upregulated in *GhWRKY33*-downregulated plants, which makes sense for enhancing whitefly resistance in *WRKY33*-downregulated plants. *HSP* proteins were previously reported as having a role in spreading geminivirus post-whitefly-infestation [40]. In *WRKY25*-downregulated plants, *HSP20* was found slightly upregulated, while in *WRKY33*-downregulated plants, its expression was found significantly increased. This shows *WRKY33* acts as a suppressor of HSP20, and silencing of *HSP20* might also be necessary for more stable whitefly resistance. Ethylene responsive factor 1 (*ERF1*) was found upregulated in whitefly tolerant varieties, and its upregulation reduced whitefly performance and enhanced Jasmonic acid signaling [41] in *GhWRKY33*-downregulated plants. *ERF1* was found upregulated remarkably, which totally justifies the accomplishment of whitefly resistance. Another gene, *JAZ1*, which was previously reported as a suppressor of the JA signaling pathway [42] was found upregulated in *GhWRKY33*-downregulated plants, which means that for a more sustainable resistance to whitefly, this downstream gene should also be targeted. Findings of our study have shown involvement of *GhWRKY25* and *GhWRKY33* as negative regulators in drought and whitefly stress in cotton. These findings suggest the exploitation of these genes in genome editing for the development of crops with improved environmental resilience.

## Figures and Tables

**Table 1:** Primer sequences used for the amplification of the targeted region.

Primers Name	Primer Sequence	Ta °C	Amplicon Size (bp)
W25-GhV-F	GCCGGAATTCAAACCATGTCGTCTCATTCG	54 °C	293
W25-GhV-R	GCCTGGTACCAGGGTTCCCTTTCACCACTT		
W33-GhV-F	GCCGGAATTCGACATCCTGGGAGCCAAATA	54 °C	283
W33-GhV-R	5′-GCCTGGTACCTGACCCTTAGGCCTTTT		
MPK6-Gh-F	GCTGAGTTGGGGTTTTTGAATG	54 °C	194
MPK6-Gh-R	GATGATAGGTAAGGATGAGCTAGTGC		
HSP20-Gh-F	TCACATCGTTTCCTTCACTTTCC	54 °C	135
HSP20-Gh-R	TCACATCGTTTCCTTCACTTTCC		
WRKY40-Gh-F	ACCATGCACGCCCTTCTCC	54 °C	139
WRKY40-Gh-R	CCGTCCCCATACCCCTCTG		
ERF1-Gh-F	GCTCAAAAGCTAATAATGAAGGGG	54 °C	147
ERF1-Gh-R	AGTATCAAAGGTTCCAAGCCAAA		
JAZ1-Gh-F	TTATGGTGGACGAGTGATTGTGTT	54 °C	131
JAZ1-Gh-R	TTGATTGGACTTCTGGCTATGCT		

**Table 2:** Promotor analysis of *GhWRKY25* and *GhWRKY33*.

Function	Motif Name	Motif Sequence	*WRKY25*	*WRKY33*
Abscisic acid responsiveness	ABRE	ACGTG	0	2
A cis-acting regulatory element essential for the anaerobic induction	ARE	AAACCA	0	1
Part of a conserved DNA module involved in light responsiveness	ATCT-motif	AATCTAATCC	0	1
Common cis-acting elements in promoter and enhancer regions	CAAT-box	CAAT	21	15
Cis-acting regulatory element related to meristem expression	CAT-box	GCCACT	0	1
Erases cellular memories of developmental age	DRE1	ACCGAGA	0	1
The estrogen-responsive element	ERE	ATTTCATA	2	6
Cis-acting regulatory element involved in light responsiveness	G-Box	CACGTT		2
Light-responsive element	GT1-motif	GGTTAA	0	1
Induction of apoptosis	MYC	CATTTG	1	2
Regulator of responses	Myb	TAACTG	1	0
The binding site for transcription factors to regulate cellular responses	STRE	AGGGG	2	2
Core promoter element around −30 of transcription start	TATA-Box	TATA	29	33
Gibberellin-responsive element	P-Box	CCTTTTG	2	0
Cis-acting element involved in salicylic acid responsiveness	TCA-element	CCATCTTTTT	0	1
WRKY TFs bind to regulate gene expression under different stress conditions	W-box	TTGACC	0	2
Part of a conserved DNA module involved in light responsiveness	Box-4	ATTAAT	3	1

## Data Availability

Not applicable.

## Supplementary Materials

The following supporting information can be downloaded at: https://www.mdpi.com/article/10.3390/genes14010171/s1, Table S1. Parameters of WRKY proteins in *G. hirsutum*.
