# Supplementary material for: Genome-Wide Analysis of WRKY Gene Family and Negative Regulation of GhWRKY25 and GhWRKY33 Reveal Their Role in Whitefly and Drought Stress Tolerance in Cotton
Source: Genes (Basel). 2023 Jan 7;14(1):171. doi: 10.3390/genes14010171 (PMC9859137; doi:10.3390/genes14010171)
Supplement: Supplementary file 1 [file genes-14-00171-s001.zip › genes-2104278-supplementary.pdf]

genes

MDPI

Genome-Wide Analysis of WRKY Gene Family and Negative Regulation of *GhWRKY25* and *GhWRKY33* Reveal Their Role in Whitefly and Drought Stress Tolerance in Cotton

Aiman Ehsan, Rubab Zahra Naqvi, Maryam Azhar, Muhammad Jawad Akbar Awan, Imran Amin, Shahid Mansoor and Muhammad Asif *

Agricultural Biotechnology Division, National Institute for Biotechnology and Genetic Engineering (NIBGE), College of Pakistan Institute of Engineering and Applied Sciences (PIEAS), Jhang Road, Faisalabad 38000, Pakistan
* Correspondence: asif.biosafety@gmail.com

Supplementary Table S1. Parameters of WRKY proteins in *G. hirsutum*.

Name	ID	Group	PI	M/W	No. of amino acids	Instability index	Aliphatic index	Hydropathicity
WRKY3	KF669771.1	I	8.47	56340.82	516	57.26	61.30	-0.766
WRKY8	KJ825858.1	I	8.40	35901.47	325	44.19	49.48	-0.970
WRKY9	KJ825859.1	I	6.76	47780.95	437	55.42	48.65	-0.931
WRKY10	KP202850.1	I	7.70	42557.49	725	60.07	56.77	-0.775
WRKY18	KF669858.1	I	6.98	62666.43	573	55.46	48.18	-0.914
WRKY22	KF669763.1	I	6.21	44373.19	402	45.39	67.79	-0.741
WRKY25	JF899343.1	I	8.47	56349.83	516	56.89	61.30	-0.765
WRKY36	KF031083.1	I	9.16	51359.16	466	48.61	57.10	-0.904
WRKY40	KC414679.1	I	8.10	52920.32	314	47.49	61.46	-0.752
WRKY44	KF031089.1	I	8.80	51396.13	275	66.01	43.27	-0.946
WRKY46	KF669766.1	I	7.26	44236.18	404	45.91	64.58	-0.836
WRKY62	KF031100.1	I	5.71	78975.12	725	60.57	56.63	-0.785
WRKY96	KF669769.1	I	7.58	55287.66	503	43.95	47.48	-0.850
WRKY97	KF669852.1	I	9.10	51431.23	466	49.47	57.10	-0.911
WRKY105	KF669770.1	I	7.65	43069.71	401	58.54	56.96	-0.756
WRKY108	KF669765.1	I	7.60	49415.80	454	53.56	55.46	-0.785
WRKY1	KF031069.1	II-a	8.45	33937.91	524	41.00	66.15	0.772
WRKY24	KF669764.1	II-a	8.68	28318.70	725	60.26	57.59	-0.770
WRKY30	KF031079.1	II-a	6.74	30502.04	277	54.48	51.84	-0.722
WRKY71	KF669857.1	II-a	8.41	34234.43	313	48.47	68.88	-0.650
WRKY73	KF031104.1	II-a	8.64	33737.86	301	63.20	49.50	-0.987
WRKY83	KF669836.1	II-a	6.46	34041.12	312	45.62	66.63	-0.588
WRKY4	KF669822.1	II-b	5.88	60612.12	561	41.31	64.76	-0.655

WRKY6	KF669821.1	II-b	8.40	38334.02	362	52.44	59.14	-0.533
WRKY16	KF669824.1	II-b	6.84	57800.24	525	40.89	65.83	-0.791
WRKY20	KJ825865.1	II-b	6.22	59094.35	545	47.33	61.45	-0.661
WRKY51	KF669825.1	II-b	8.57	54526.58	506	50.88	72.94	-0.488
WRKY76	KF669827.1	II-b	7.28	61261.46	564	50.04	61.26	-0.694
WRKY79	KF669828.1	II-b	7.03	55519.33	508	62.38	61.28	-0.636
WRKY81	KF669830.1	II-b	5.85	48999.44	442	50.67	64.68	-0.799
WRKY87	KF669829.1	II-b	7.24	60972.64	561	47.08	61.76	-0.661
WRKY90	KF669851.1	II-b	6.77	54461.88	503	54.63	63.88	-0.660
WRKY2	KF669759.1	II-c	9.64	18659.32	161	42.99	55.71	-0.802
WRKY14	KF669762.1	II-c	9.22	18571.00	570	52.41	64.37	-0.670
WRKY15	KF669833.1	II-c	9.64	17366.69	520	34.73	35.58	0.741
WRKY23	KX184202.1	II-c	5.94	35610.34	315	66.70	51.65	-0.883
WRKY37	KF031084.1	II-c	7.77	34236.37	313	50.70	63.80	-0.612
WRKY38	KF669838.1	II-c	8.82	105978.61	953	39.84	78.79	-0.478
WRKY49	KF669813.1	II-c	8.99	21522.30	189	52.00	64.97	-0.829
WRKY52	KF669850.1	II-c	9.81	26090.86	224	48.37	50.89	-0.721
WRKY67	KF669844.1	II-c	6.07	33400.90	309	59.26	53.01	-0.739
WRKY68	KJ551845.1	II-c	8.71	33186.32	295	65.23	59.83	-0.804
WRKY74	KF031105.1	II-c	6.27	35524.15	507	54.64	59.84	-0.784
WRKY75	MH138002.1	II-c	9.30	19510.07	170	32.05	60.12	-0.831
WRKY77	KF669816.1	II-c	8.17	19833.50	171	40.91	60.47	-0.912
WRKY80	KF031106.1	II-c	6.21	35684.65	466	48.75	63.88	-0.615
WRKY86	KF669817.1	II-c	9.62	17564.10	150	70.27	48.73	-0.895
WRKY92	KF669849.1	II-c	6.83	35621.36	314	59.37	40.41	-0.961
WRKY94	KF669847.1	II-c	9.47	18536.90	160	29.25	57.19	-0.955
WRKY95	KF669855.1	II-c	6.26	39610.00	360	65.03	58.33	-0.771
WRKY98	KF669818.1	II-c	5.91	27273.48	238	53.73	67.18	-0.936
WRKY100	KF031114.1	II-c	7.65	35926.41	320	52.49	66.38	-0.666
WRKY103	KF031115.1	II-c	6.80	31552.14	467	58.77	64.58	-0.531
WRKY104	KF031116.1	II-c	6.26	36085.12	321	63.73	54.61	-0.778
WRKY112	KF669803.1	II-c	5.06	18401.12	157	54.39	49.62	-1.186
WRKY113	KF669804.1	II-c	7.69	23538.15	207	39.87	54.15	-0.875
WRKY114	KF669805.1	II-c	4.98	18723.77	162	55.46	54.75	-0.906
WRKY115	KF669839.1	II-c	6.43	19887.05	174	58.48	52.07	-0.973
WRKY116	KF031118.1	II-c	8.88	25436.78	489	60.28	56.48	-0.693
WRKY11	KJ825861.1	II-d	9.48	36298.14	335	45.24	64.96	-0.459
WRKY12	KF669853.1	II-d	9.51	36134.00	335	47.12	64.09	-0.447
WRKY21	KT983420.1	II-d	9.51	36874.78	331	55.98	62.78	-0.768
WRKY32	KF031081.1	II-d	9.73	38735.93	358	57.80	62.40	-0.698
WRKY39	KF220643.1	II-d	4.88	293756.35	323	56.47	58.85	-0.876

WRKY42	KF669797.1	II-d	9.40	37841.67	345	49.52	62.20	-0.653
WRKY47	KF669798.1	II-d	9.66	37240.19	336	58.05	61.55	-0.734
WRKY54	KF031095.1	II-d	9.92	36662.26	504	48.24	66.45	-0.529
WRKY69	KF669845.1	II-d	9.36	36599.47	330	47.01	63.24	-0.614
WRKY78	KF669801.1	II-d	9.66	38721.90	354	46.65	66.69	-0.588
WRKY84	KF669802.1	II-d	9.59	36688.47	341	45.61	66.63	-0.447
WRKY93	KF669854.1	II-d	9.58	39324.46	358	58.34	61.31	-0.711
WRKY111	KF031117.1	II-d	9.51	36874.78	355	50.20	64.28	-0.738
WRKY118	KF031119.1	II-d	9.51	36846.73	331	56.50	62.21	-0.775
WRKY19	KF669784.1	II-e	9.25	30225.60	264	53.69	74.92	-0.597
WRKY26	KJ825868.1	II-e	6.51	35568.21	325	49.29	48.00	-0.715
WRKY28	KF669796.1	II-e	5.63	38863.39	292	65.23	71.06	-0.524
WRKY29	KF669795.1	II-e	6.71	35537.24	117	34.32	120.60	0.150
WRKY31	KF031080.1	II-e	5.06	30088.36	561	50.29	61.09	-0.690
WRKY43	KF031088.1	II-e	5.15	30485.88	276	46.69	52.64	-0.806
WRKY48	JN609590.1	II-e	5.74	35950.67	360	65.03	58.33	-0.771
WRKY53	KF669786.1	II-e	5.12	29943.25	267	56.52	59.85	-0.928
WRKY57	KF669787.1	II-e	5.30	37321.46	349	42.63	55.13	-0.768
WRKY58	KF031097.1	II-e	5.45	42389.22	560	45.05	62.79	-0.682
WRKY61	KF669790.1	II-e	5.60	34014.05	300	47.60	62.37	-0.733
WRKY65	KF031102.1	II-e	5.06	25801.60	251	54.22	52.83	-0.925
WRKY72	KF669791.1	II-e	4.95	49320.61	459	55.61	58.95	-0.581
WRKY82	KF669768.1	II-e	5.14	40557.28	371	58.41	53.58	-1.040
WRKY85	KF669792.1	II-e	5.15	30485.88	276	46.69	52.64	-0.806
WRKY91	KF669793.1	II-e	5.13	29816.79	273	60.12	47.18	-0.792
WRKY5	KF669781.1	III	5.63	38298.01	335	50.80	65.46	-0.693
WRKY7	KF669776.1	III	5.97	34664.65	305	51.44	61.97	-0.847
WRKY13	KJ825862.1	III	7.09	23054.84	203	46.51	66.26	-0.865
WRKY17	KJ825864.1	III	9.11	24545.83	212	44.65	58.40	-0.951
WRKY27	KJ825869.1	III	5.41	34657.48	305	52.68	60.10	-0.830
WRKY33	KC414677.1	III	5.10	40785.09	510	45.80	45.31	-0.843
WRKY34	KF031082.1	III	5.61	35311.46	354	47.53	65.85	-0.591
WRKY41	KF031087.1	III	5.46	40062.62	350	47.15	68.26	-0.463
WRKY50	KF669783.1	III	5.58	37405.83	334	49.20	68.56	-0.562
WRKY56	KF031096.1	III	6.18	36738.61	335	50.80	65.46	-0.693
WRKY59	KF031098.1	III	5.33	32150.71	349	55.24	67.42	-0.515
WRKY60	KF669778.1	III	5.42	39792.12	355	49.12	65.38	-0.724
WRKY64	KF031101.1	III	6.21	22645.47	355	52.75	63.69	-0.730
WRKY70	MF278614.1	III	5.97	34648.65	305	50.81	62.30	-0.838
WRKY88	KF031110.1	III	5.21	31214.79	335	45.55	56.54	-0.584
WRKY101	KF669777.1	III	6.04	39334.71	360	56.15	55.86	-0.682

WRKY102	KF669772.1	III	5.57	34311.27	302	62.74	56.56	-0.751
WRKY106	KF669780.1	III	6.79	35027.96	305	51.67	53.34	-0.963

PI=Isoelectric point, M/W=Molecular weight
